# Re-emergence of dengue, chikungunya, and Zika viruses in 2021 after a 10-year gap in Gabon

**DOI:** 10.1016/j.ijregi.2022.08.013

**Published:** 2022-09-05

**Authors:** Yuri Ushijima, Haruka Abe, Marien J.V.M. Mbadinga, Georgelin Nguema Ondo, Rodrigue Bikangui, Selidji T. Agnandji, Bertrand Lell, Jiro Yasuda

**Affiliations:** a Department of Emerging Infectious Diseases, Institute of Tropical Medicine (NEKKEN), Nagasaki University, Nagasaki, Japan; b Centre de Recherches Médicales de Lambaréné, Lambaréné, Gabon; c Institute for Tropical Medicine, University of Tübingen, Tübingen, Germany; d Division of Infectious Diseases and Tropical Medicine, Medical University of Vienna, Vienna, Austria; e Department of Emerging Infectious Diseases, National Research Center for the Control and Prevention of Infectious Diseases (CCPID), Nagasaki University, Nagasaki, Japan; f Graduate School of Biomedical Sciences, Nagasaki University, Nagasaki, Japan

**Keywords:** dengue virus, chikungunya virus, Zika virus, Gabon, Africa, phylogeny

## Abstract

Mosquito-borne viral infections are a major concern in endemic areas, such as Africa. Although outbreaks have been reported throughout Africa, only a few surveillance studies have been conducted in Gabon since the outbreaks of dengue virus (DENV) and chikungunya virus (CHIKV) in 2010. Therefore, the current situation is unknown. This study aimed to investigate the presence of arboviruses, especially DENV (serotypes 1–4), CHIKV, and Zika virus (ZIKV), in Gabon, Central Africa. Between 2020 and 2021, we collected 1060 serum samples from febrile patients and screened them against viruses using reverse transcription-quantitative PCR. We detected two DENV serotypes 1 (DENV-1), one CHIKV, and one ZIKV, and subsequently analyzed the genome sequences. To determine the genetic diversity and transmission route of the viruses, phylogenetic analysis was performed using complete or partial genome sequences. The DENV-1 and CHIKV strains detected in this study were closely related to the previous Gabonese strains, whereas the recent ZIKV strain was genetically different from a strain detected in 2007 in Gabon. This study provides new genomic information on DENV-1, CHIKV, and ZIKV that were detected in Gabon and insight into the circulation of the viruses in the country and their introduction from neighboring African countries.

## Highlights

• DENV-1, CHIKV, and ZIKV were detected in Gabon in 2021.
• DENV appeared to switch to serotype 1 from serotype 2 and 3 since 2010.
• *Aedes albopictus*‒adapted CHIKV appears to circulate repeatedly in Central Africa.
• The recent Gabonese ZIKV strain was genetically different from the previous strain.

## Introduction

Infections by mosquito-borne viruses, including dengue virus (DENV), chikungunya virus (CHIKV), and Zika virus (ZIKV), are caused primarily by the bite of *Aedes* mosquitoes infected with these viruses (Gubler, 2001). Outbreaks of arboviral diseases have been consistently reported in Africa and the disease burden is increasing (Wilder-Smith et al., 2017; Africa CDC, 2022). Recent examples were outbreaks of chikungunya in the Democratic Republic of the Congo in 2019 and dengue in Burkina Faso in 2017. Sporadic molecular and serological surveys have been conducted on DENV, CHIKV, and ZIKV in Gabon since the first documentation of the outbreaks or occurrence of infectious diseases caused by DENV-2, CHIKV, and ZIKV in 2007 (Leroy et al., 2009; Grard et al., 2014; Abe et al., 2020; Lim et al., 2021; Ushijima et al., 2021). However, little information is available on the genetic diversity and spatiotemporal dynamics of these viruses. Therefore, we investigated the recent situation by analyzing the genomes of these three viruses detected in febrile patients around Lambaréné in Gabon, 2020‒2021.

## Methods

A total of 1060 serum samples were collected from febrile patients who visited Albert Schweitzer Hospital between 2020 and 2021 in Lambaréné, Gabon. RNAs were extracted from the samples and screened for DENV, CHIKV, and ZIKV genes by reverse transcription-quantitative PCR (RT-qPCR). RT-PCR was subsequently conducted on the positive samples to amplify the target viral genes. To characterize genetic diversity with high resolution, next-generation sequencing was performed to identify the whole-genome sequence. Phylogenetic analysis was performed using the sequences obtained (Supplementary Methods).

## Results and Discussion

Of the 1060 samples, two (SYMAV-H0408, two-year-old female in Lambaréné; SYMAV-H0983, 29-year-old female in Lambaréné), one (SYMV-H0915, 6-year-old male in Sindara), and one (SYMAV-H0931, 12-year-old male in Ndjolé) were positive for DENV-1, CHIKV, and ZIKV, respectively, as analyzed by RT-qPCR. All patients visited the hospital 2‒3 days after the onset of fever and presented with a body temperature of 38°C or higher, but it was difficult to diagnose them with specific clinical manifestations.

**Figure 1:**
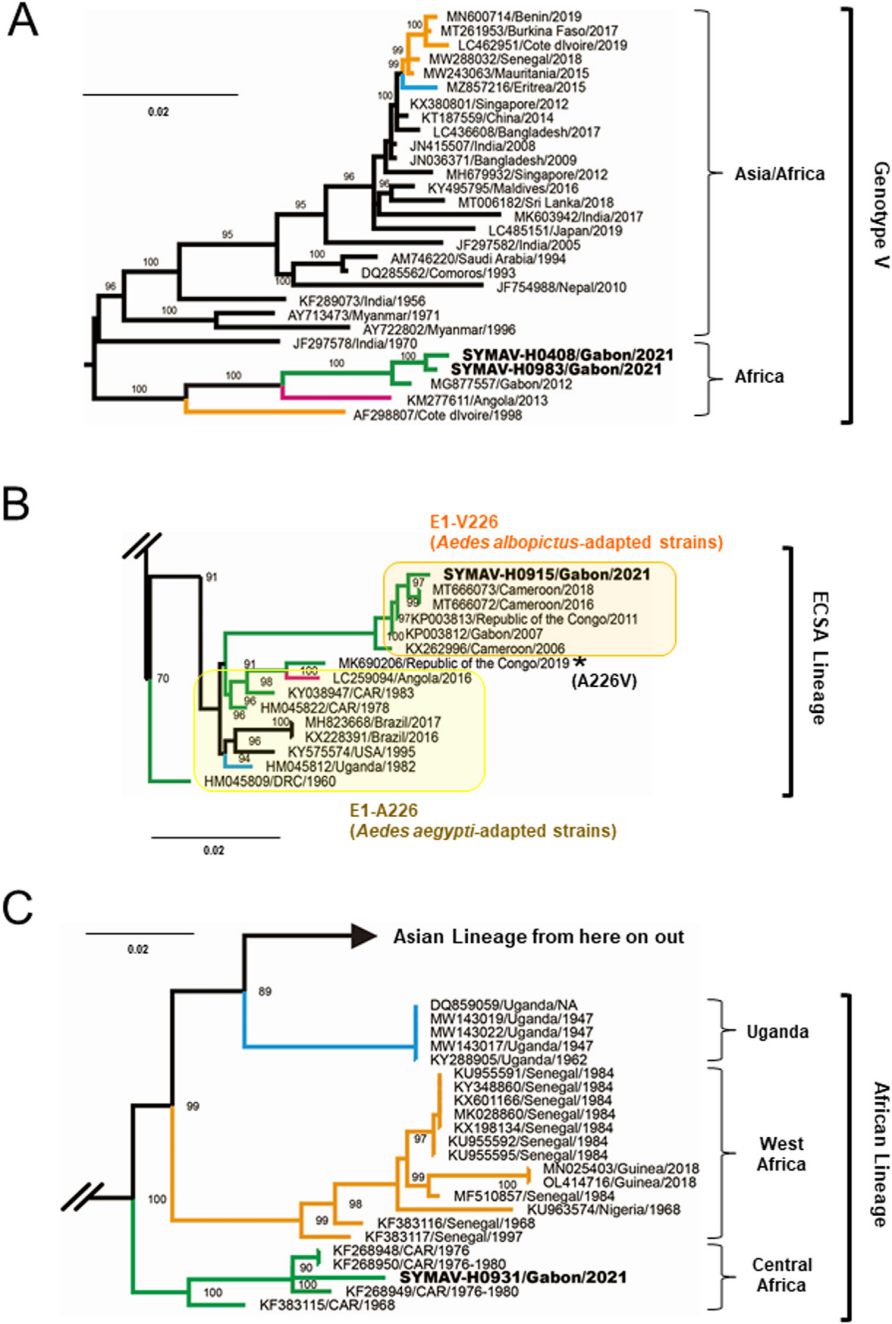
Phylogenetic analysis of full or near full-length gene. (A) Envelope of dengue virus serotype 1 (1485 bp), (B) Envelope 1 of chikungunya virus (1295 bp), and (C) Non-structural protein 3 of Zika virus (1851 bp). A maximum-likelihood tree was inferred with 1000 bootstrap replicates. Bootstrap values of ≥70% are shown at the main nodes. Virus genotypes and lineages are shown on the right. The Gabonese strains detected in this study are shown in bold. The asterisk in Figure 1B indicates the previous Republic of the Congo strain detected in 2019. Colours represent lineages of African strain: green, Central Africa; orange, West Africa; blue, East Africa; and pink, South Africa. Scale bars indicate nucleotide substitutions per site. An entire illustration of phylogeny of each virus, including strains in the world, is provided in Supplementary Figure S2.

To investigate the phylogeny of the detected viruses, we determined the sequences of the DENV-1 envelope (E) gene from two samples, and the complete genome sequence was determined for SYMAV-H0983 with a Ct value of 16.7 (GenBank accession nos. **LC707378** and **LC707382**). Phylogenetic analysis inferred that these two samples belonged to the African group of genotype V and were closely related to the Gabon/2012 strain (Figure 1A and Supplemental Figure S1). The phylogenetic tree also showed that the clade of the Gabonese strains was separated from that of the Angola/2013 strain, suggesting that DENV-1 has circulated persistently in Gabon since its last appearance. Considering previous reports from 2015‒2017 showing the detection of both DENV-2 and 3 in the same area (Abe et al., 2020; Lim et al., 2021), the serotype has likely now switched to DENV-1. The emergence of severe dengue cases should be carefully monitored in the area because secondary infections by other serotypes increase the risk of developing severe dengue (Vaughn et al., 2000).

A phylogenetic tree of the CHIKV envelope 1 (E1) gene (GenBank accession no. **LC707379**) revealed that SYMAV-H0915 belonged to the East/Central/South African (ECSA) lineage and fell within the same group as the Cameroon/2018 and Gabon/2007 strains harboring E1-A226V, which confers a fitness advantage of CHIKV in *Aedes albopictus* (Tsetsarkin et al., 2007) (Figure 1B). Bayesian phylogeny suggested that the recent Gabonese CHIKV strain was branched off from the strain detected in the Republic of Congo in 2011 and diverged earlier than the Cameroon isolates in 2016 and 2018 (Supplemental Figure S3). CHIKV appeared to circulate repeatedly in Cameroon, Gabon, and the Republic of the Congo. The CHIKV detected in the Republic of the Congo in 2019 exhibited the same E1-A226V mutation as the Gabonese strains, suggesting a vector-host switch event from *Aedes aegypti* to *Aedes albopictus* (Fritz et al., 2019). Therefore, the spread of a new strain with A226V mutation could be a threat to Gabon and other *Ae. albopictus*‒dominated regions (Kraemer et al., 2019).

**Figure 2:**
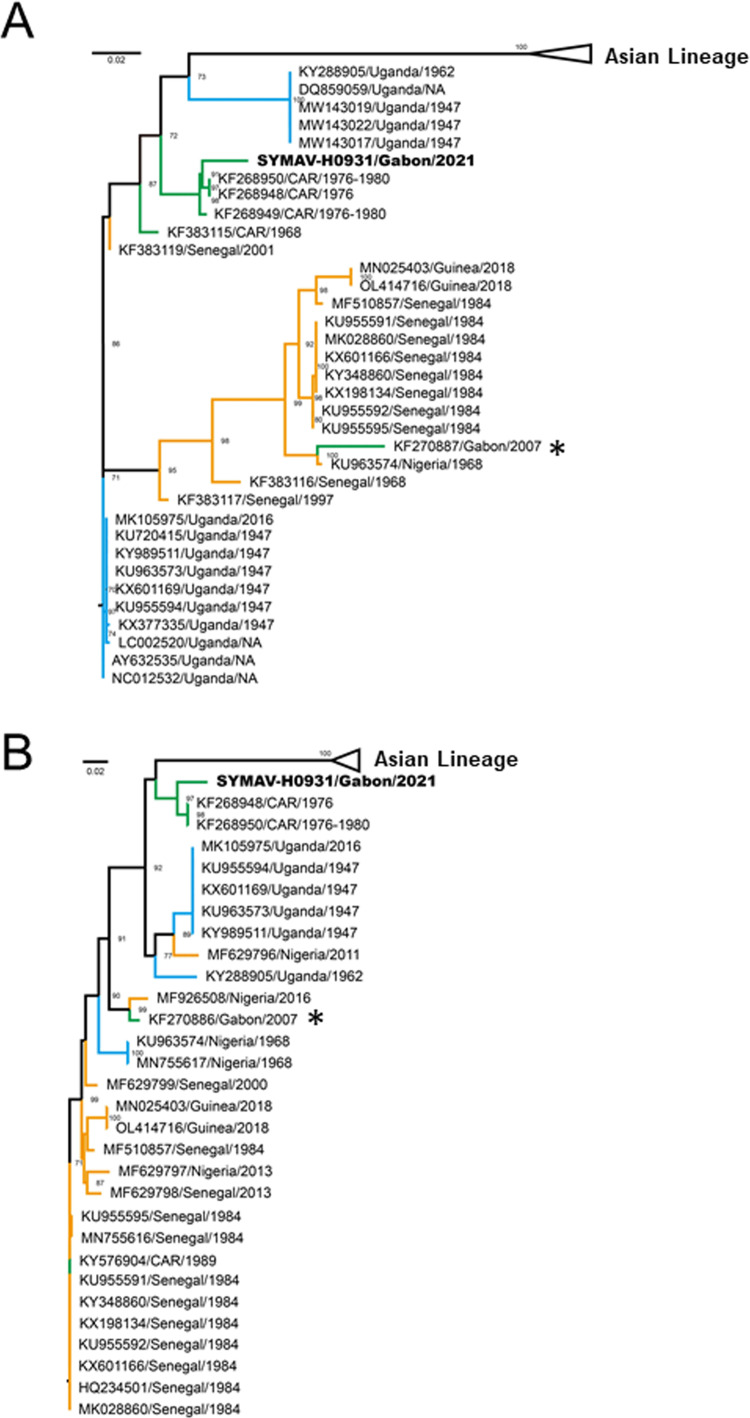
Phylogenetic analysis of partial-length of (A) non-structural protein 3 (772 bp) and (B) envelope (750 bp) of Zika virus to compare genetic diversity with the strain detected in 2007 in Gabon. A maximum-likelihood tree was inferred with 1000 bootstrap replicates. Bootstrap values of ≥70% are shown at the nodes. For better visualization of sequence positions, Asian lineages were collapsed and shown as triangles. The Gabonese strain detected in this study is shown in bold. The asterisk indicates the previous Gabonese strain detected in 2007. Colours represent lineages of African strain: green, Central Africa; orange, West Africa; and blue, East Africa. Scale bars indicate nucleotide substitutions per site.

In Gabon, ZIKV was only reported in 2007, and its genome information was very limited, except for partial sequences of the E and non-structural 3 (NS3) genes from one strain (Grard et al., 2014). In this study, the sequences of the full-length NS3 gene and 1,065 bp E genes of SYMAV-H0931 were determined (GenBank accession nos. **LC707380** and **LC707381**). A phylogenetic tree using the NS3 gene showed that the strain belonged to the African lineage and was located in the Central African clade, which contained old strains detected in the 1960‒1980s (Figure 1C), unlike the previous Gabonese strain belonging to the West African clade (Figure 2A and B). These results indicate that the recent ZIKV has either been newly introduced after 2007 or the traditional strain present in Central Africa since 1970–1980s has been circulating in Gabon for a long time without detection.

In conclusion, this study revealed ongoing DENV-1, CHIKV, and ZIKV circulation around Lambaréné and Gabon (Supplemental Figure S6). Considering that these viral infections are often subclinical, requiring no hospital visit, and that the preferred habitat (urban, rural, and so on) varies with the type of mosquito, active surveys based on community-based samples and expanded study areas will help understand the entire country's situation.

## Appendix Supplementary materials

Image, application 1

## References

[bib0001] Abe H, Ushijima Y, Loembe MM, Bikangui R, Nguema-Ondo G, Mpingabo PI, Zadeh VR, Pemba CM, Kurosaki Y, Igasaki Y, de Vries SG, Grobusch MP, Agnandji ST, Lell B, Yasuda J. (2020). Re-emergence of dengue virus serotype 3 infections in Gabon in 2016-2017, and evidence for the risk of repeated dengue virus infections. Int J Infect Dis.

[bib0002] Africa CDC, Diseases. https://africacdc.org/disease/(accessed 22 August 2022).

[bib0003] Fritz M, Taty RT, Portella C, Guimbi C, Mankou M, Leroy EM, Becquart P. (2019). Re-emergence of chikungunya in the Republic of the Congo in 2019 associated with a possible vector-host switch. Int J Infect Dis.

[bib0004] Grard G, Caron M, Mombo IM, Nkoghe D, Mboui Ondo S, Jiolle D, Fontenille D, Paupy C, Leroy EM (2014). Zika virus in Gabon (Central Africa)–2007: a new threat from Aedes albopictus?. PLoS Negl Trop Dis.

[bib0005] Leroy EM, Nkoghe D, Ollomo B, Nze-Nkogue C, Becquart P, Grard G, Pourrut X, Charrel R, Moureau G, Ndjoyi-Mbiguino A, De-Lamballerie X. (2009). Concurrent chikungunya and dengue virus infections during simultaneous outbreaks, Gabon, 2007. Emerg Infect Dis.

[bib0006] Lim JK, Fernandes JF, Yoon IK, Lee JS, Mba RO, Lee KS, Namkung S, Yang JS, Bae SH, Lim SK, Lell B, Esen M, Loembe MM, Kremsner PG, Alexander N, Agnandji ST. (2021). Epidemiology of dengue fever in Gabon: Results from a health facility-based fever surveillance in Lambaréné and its surroundings. PLoS Negl Trop Dis.

[bib0007] Gubler DJ. (2001). Human Arbovirus Infections Worldwide. Ann N Y Acad Sci.

[bib0008] Kraemer MUG, Reiner RC, Brady OJ, Messina JP, Gilbert M, Pigott DM, Yi D, Johnson K, Earl L, Marczak LB, Shirude S, Davis Weaver N, Bisanzio D, Perkins TA, Lai S, Lu X, Jones P, Coelho GE, Carvalho RG, Van Bortel W, Marsboom C, Hendrickx G, Schaffner F, Moore CG, Nax HH, Bengtsson L, Wetter E, Tatem AJ, Brownstein JS, Smith DL, Lambrechts L, Cauchemez S, Linard C, Faria NR, Pybus OG, Scott TW, Liu Q, Yu H, Wint GRW, Hay SI, Golding N (2019). Past and future spread of the arbovirus vectors Aedes aegypti and Aedes albopictus. Nat Microbiol.

[bib0009] Tsetsarkin KA, Vanlandingham DL, McGee CE (2007). Higgs S. A single mutation in chikungunya virus affects vector specificity and epidemic potential. PLoS Pathog.

[bib0010] Ushijima Y, Abe H, Nguema Ondo G, Bikangui R, Massinga Loembé M, Zadeh VR, Essimengane JGE, Mbouna AVN, Bache EB, Agnandji ST, Lell B, Yasuda J (2021). Surveillance of the major pathogenic arboviruses of public health concern in Gabon, Central Africa: increased risk of West Nile virus and dengue virus infections. BMC Infect Dis.

[bib0011] Vaughn DW, Green S, Kalayanarooj S, Innis BL, Nimmannitya S, Suntayakorn S, Endy TP, Raengsakulrach B, Rothman AL, Ennis FA, Nisalak A. (2000). Dengue viremia titer, antibody response pattern, and virus serotype correlate with disease severity. J Infect Dis.

[bib0012] Wilder-Smith A, Gubler DJ, Weaver SC, Monath TP, Heymann DL, Scott TW (2017). Epidemic arboviral diseases: priorities for research and public health. Lancet Infect Dis.

