# Supplementary material for: Re-emergence of dengue, chikungunya, and Zika viruses in 2021 after a 10-year gap in Gabon
Source: IJID Reg. 2022 Sep 5;5:68–71. doi: 10.1016/j.ijregi.2022.08.013 (PMC9529494; doi:10.1016/j.ijregi.2022.08.013)
Supplement: Supplementary file 1 [file mmc1.docx]

**APPENDIX**

Re-emergence of dengue, chikungunya, and Zika viruses in 2021 after a 10-year gap in Gabon

Yuri Ushijima^a^, Haruka Abe^a^, Marien J.V.M. Mbadinga^b^, Georgelin Nguema Ondo^b^, Rodrigue Bikangui^b^, Selidji T. Agnandji^b,c^, Bertrand Lell^b,c,d^, Jiro Yasuda^a,e,f,*^

^a^ *Department of Emerging Infectious Diseases, Institute of Tropical Medicine (NEKKEN), Nagasaki University, Nagasaki, Japan*

^b^ *Centre de Recherches Médicales de Lambaréné, Lambaréné, Gabon*

^c^ *Institute for Tropical Medicine, University of Tübingen, Tübingen, Germany*

^d^ *Division of Infectious Diseases and Tropical Medicine, Medical University of Vienna, Vienna, Austria*

^e^ *Department of Emerging Infectious Diseases, National Research Center for the Control and Prevention of Infectious Diseases (CCPID), Nagasaki University, Nagasaki, Japan*

^f^ *Graduate School of Biomedical Sciences, Nagasaki University, Nagasaki, Japan*

^*^ Corresponding author:

Jiro Yasuda

Department of Emerging Infectious Diseases, National Research Center for the Control and Prevention of Infectious Diseases (CCPID), Nagasaki University

1-12-4 Sakamoto, Nagasaki 852-8523, Japan.

*E-mail address:* j-yasuda@nagasaki-u.ac.jp

**Keywords**

dengue virus; chikungunya virus; Zika virus; Gabon; Africa; phylogeny

**Supplemental Methods**

**Viral RNA extraction and detection by reverse transcription-quantitative polymerase chain reaction PCR (RT-qPCR)**

Viral RNA was extracted from 140 μL of each serum sample with a QIAamp Viral RNA Mini Kit (Qiagen, Hilden, Germany) according to the manufacturer’s instructions. RT-qPCR was performed in a 20 μL reaction using a One Step PrimeScript III RT-qPCR Mix (Takara Bio, Shiga, Japan). Each reaction mixture contained 10 μL 2 × One Step PrimeScript RT-qPCR Mix, 0.4 μL ROX Reference Dye, 2 μL of primer and TaqMan probe mixtures, 2 μL RNA template and RNase-free water up to 20 μL. Final concentration of the primers and probes were as follows: forward and reverse primers for DENV-1 and DENV-3 (1 µM), DENV-2, DENV-4, CHIKV and ZIKV (0.5 µM), probes for each DENV (0.18 µM), and CHIKV and ZIKV (0.2 µM). The probes were labelled with 5′-FAM/BHQ-1. RT-qPCR assays were carried out with a StepOnePlus instrument (Thermo Fisher Scientific, Waltham, MA, USA) under the following conditions: 5 minutes at 52°C, 10 seconds at 95°C, and 45 cycles of 5 seconds at 95°C and 35 seconds at 60°C. The primers and probes specific for DENV were used as previously reported (Vaughn et al., 2000) and those specific for CHIKV and ZIKV were designed using the sequences conserved region after strains worldwide were aligned. The primers and probes used in this study are listed in Supplementary Table S1. Data collected from the RT-qPCR assays were analysed using software included in the StepOnePlus system. Samples reaching threshold cycle (Ct) values under 40 were set as positive.

**Sequencing of target genes of detected viruses**

Reverse transcription-PCR (RT-PCR) was conducted with PrimeScript II High-Fidelity One Step RT-PCR Kit (Takara Bio) using designed primers (Supplementary Table S1) to amplify the following target genes: envelope (E) for DENV-1, envelope 1 (E1) for CHIKV, and non-structural protein 3 (NS3) and E for ZIKV. After agarose gel purification of the desired amplicons with QIAquick Gel Extraction Kit (Qiagen, Hilden, Germany), the PCR products were processed with the BigDye Terminator v3.1 Cycle Sequencing Kit (Thermo Fisher Scientific) and analysed with a ABI3500 capillary sequencer (Thermo Fisher Scientific) to obtain sequence data. Sequenced fragments were assembled using CLC Main Workbench 8 software (Qiagen) and consensus sequences were extracted.

**Whole-genome sequencing of DENV-1 (SYMAV-H0983)**

SYMAV-H0983 RNA sample was selected for whole-genome sequencing because of the relatively high viral titer (Ct values < 30 by RT-qPCR). The viral RNAs were reverse-transcribed by SuperScript IV Reverse Transcriptase (Thermo Fisher Scientific, Waltham, MA, USA) using 11 μL of the RNA combined with random hexamers according to the manufacturer’s protocol. To efficiently sequence full-length coding regions by a next-generation sequencer, a multiplex PCR method was performed as reported previously (Abe H et al., 2021). First, primer sets for multiplex PCR were designed by the online program Primal Scheme (http://primal.zibraproject.org/) under the conditions of the amplicon size 450 bp and the overlap 50 bp. The template sequence was the Gabon/2012 strain (GenBank accession no. MG877557). Primer sets were divided into two pools as described previously (Quick et al., 2021). Multiplex PCR reaction was performed using Q5 High-Fidelity DNA Polymerase (New England Biolabs, Ipswich, MA, USA) with 25 μL mixture contained 5 μL Q5 Reaction Buffer, 0.5 μL dNTP Mixture (10 mM each), 0.25 μL Q5 High-Fidelity DNA Polymerase, 0.015 μM of each primer, 15 μL purified cDNA, and RNase-free water up to 25 μL, under the following conditions: 30 s at 98 °C; 40 cycles of 15 s at 98 °C and 4 min at 65 °C. Multiplex PCR products were purified using Agencourt AMPure XP (Beckman Coulter) and eluted in 30 μL RNase-free water, followed by quantitation with a Qubit 2.0 Fluorometer and a Qubit dsDNA HS Assay Kit (Thermo Fisher Scientific). Library was prepared using 500 ng of the multiplex PCR product and an NEBNext Ultra II FS DNA Library Prep Kit for Illumina (New England Biolabs) in combination with NEBNext Multiplex Oligos for Illumina (Dual Index Primers Set 1) (New England Biolabs) according to the manufacturer’s instructions. After quality and quantity check of the library using an Agilent 2100 Bioanalyzer (Agilent Technologies, Santa Clara, CA, USA) with a High Sensitivity DNA Kit (Agilent), sequencing was performed using a 300-cycle High Output Kit (Illumina, San Diego, CA, USA) on a MiniSeq sequencer (Illumina). Mapping of the paired-end reads was performed on CLC Genomics Workbench 11.0.1 software (Qiagen) using whole-genome sequence of the Gabon/2012 strain as a template. Consensus sequences were extracted and aligned with reference strains on BioEdit 7.0.5.3 software (http://www.mbio.ncsu.edu/BioEdit/bioedit.html).

**Phylogenetic analysis**

For phylogenetic analysis of whole-genome or partial-genome sequences, high-coverage reference sequences were obtained from the Virus Pathogen Resource (https://www.viprbrc.org/). Reference sequences were selected to cover the main clades of each virus. Consensus sequences of DENV-1, CHIKV, and ZIKV strains detected in this study and reference sequences were aligned and checked manually for gaps to be removed. Finally, each phylogenetic analysis included 62 sequences for whole-genome of DENV-1 (SYMAV-H0983, 10179 nt), 185 sequences for full-length of DENV-1 E (SYMAV-H0408 and H0983, 1485 nt), 108 sequences for near full-length of CHIKV E1 (SYMAV-H0915, 1295 nt), 105 sequences for full-length of ZIKV NS3 (SYMAV-H0931, 1851 nt), 106 sequences for partial-length of ZIKV NS3 (772 nt), and 91 sequences for partial-length of ZIKV E (750 nt). Maximum-Likelihood (ML) analysis was performed using IQ-TREE software (http://www.iqtree.org/) under the condition of a best-fit substitution model: TIM2+F+I+G4 for DENV-1 complete genome, TIM2+F+I+G4 for DENV-1 E, TIM2e+I for CHIKV E1, TN+F+G4 for ZIKV NS3, TNe+G4 for ZIKV NS3 and E partial regions. A total of 1,000 bootstrap replicates were generated in the analyses. Bayesian analysis was also performed with timestamped reference sequences that include complete gene sequences of African strains using BEAST v1.8.0 software (http://beast.community/) under the conditions of the SRD08 model, the uncorrelated relaxed lognormal clock, and 10 million (DENV-1 E) or 40 million (CHIKV E1 and ZIKV NS3) generations. For countries outside Africa, reference strains were widely selected from each continent and from various time points of the collection date. Finally, 142, 154, and 127 sequences of DENV-1, CHIKV, and ZIKV were included in the phylogenetic analysis, respectively. The effective sample size (ESS) values were verified to show more than 300. For better visualization, the phylogenetic trees generated by both ML and Bayesian analyses were modified using FigTree v1.4.2 software (<http://tree.bio.ed.ac.uk/software/figtree>).

**Graphic map**

The map was created with free software (<https://n.freemap.jp/tp/Africa>) and the information of each virus which was revealed in this study were added to the map using the Microsoft PowerPoint 2016.

**Data availability statement**

Genomic data of the newly sequenced samples were deposited in the GenBank database with the accession number LC707378‒ LC707382.

**Supplemental Figures and Tables**

**
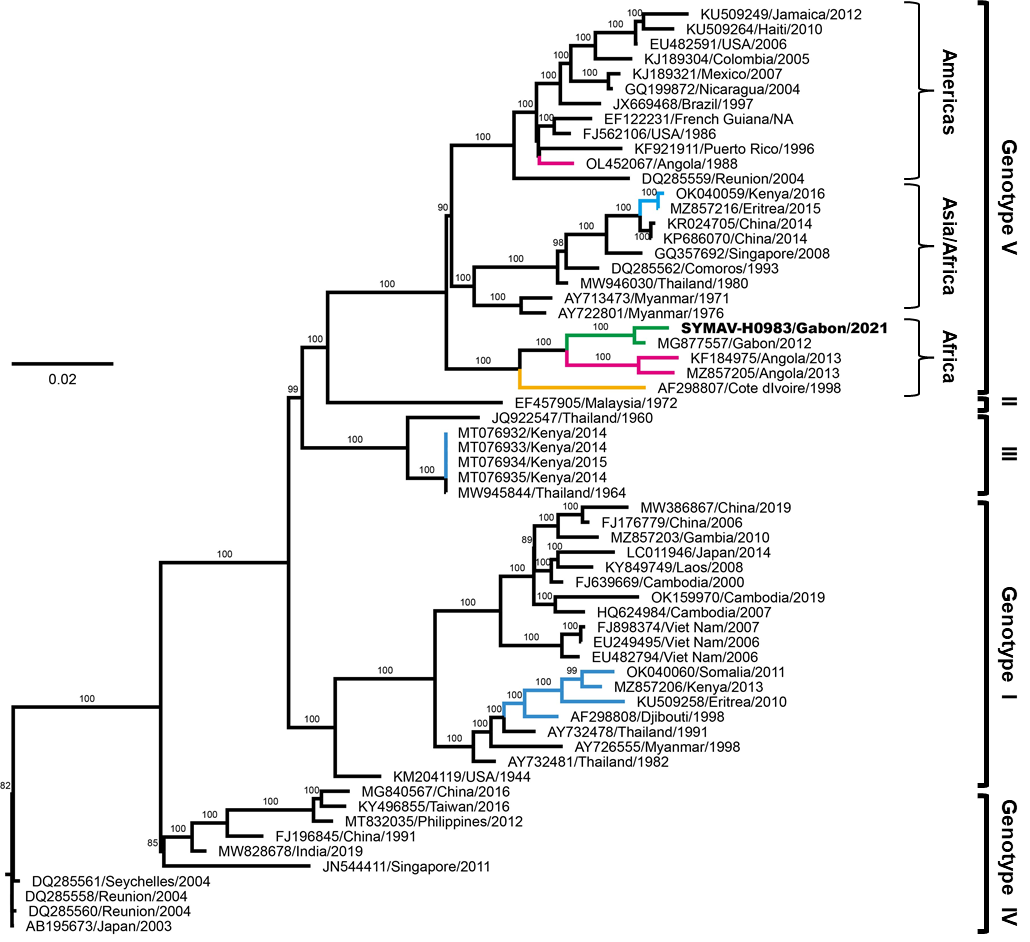
**

**Figure S1.** Phylogenetic analysis of the complete genome sequence of DENV-1 (SYMAV-H0983). A maximum-likelihood tree was inferred using all African strains available. Bootstrap values of ≥70% are shown at nodes of the tree. Virus genotypes are shown on the right. The Gabonese strain detected in this study are shown in bold. Colours represent lineages of African strain: green, Central Africa; orange, West Africa; blue, East Africa; pink, South Africa. Scale bar indicates nucleotide substitutions per site.

**
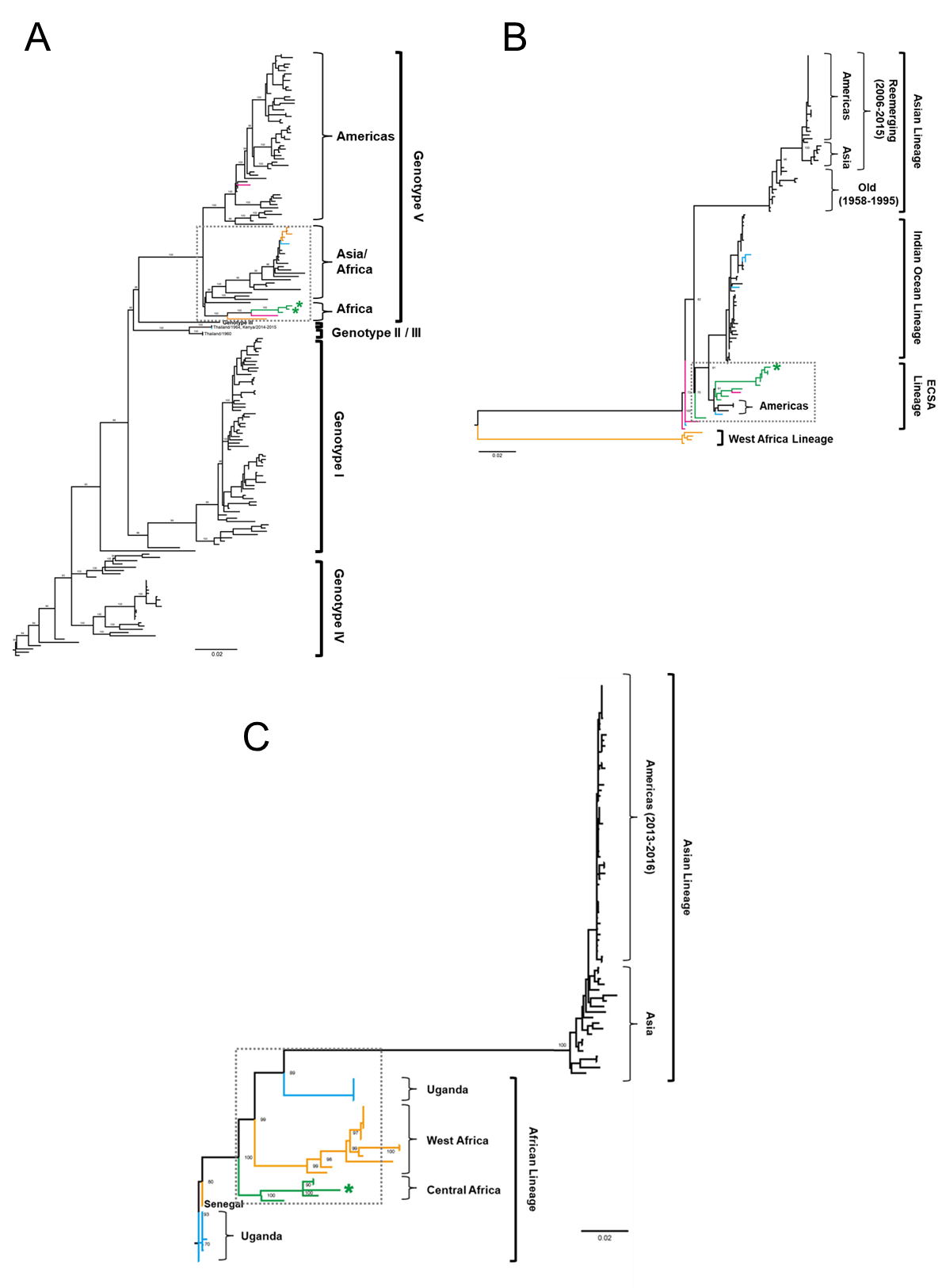
Figure S2.** Entire illustration of phylogeny including strains in the world. (A) Envelope of dengue virus serotype 1 (1485 bp), (B) Envelope 1 of chikungunya virus (1295 bp), and (C) Non-structural protein 3 of Zika virus (1851 bp). A maximum-likelihood tree was inferred with 1000 bootstrap replicates. Bootstrap values of ≥70% are shown at the main nodes. The asterisk indicates the Gabonese strain detected in this study. Colours represent lineages of African strain: green, Central Africa; orange, West Africa; blue, East Africa; pink, South Africa. Scale bars indicate nucleotide substitutions per site. An enlargement of the area enclosed by the dotted line is provided in Figure 1A‒C in the main text.


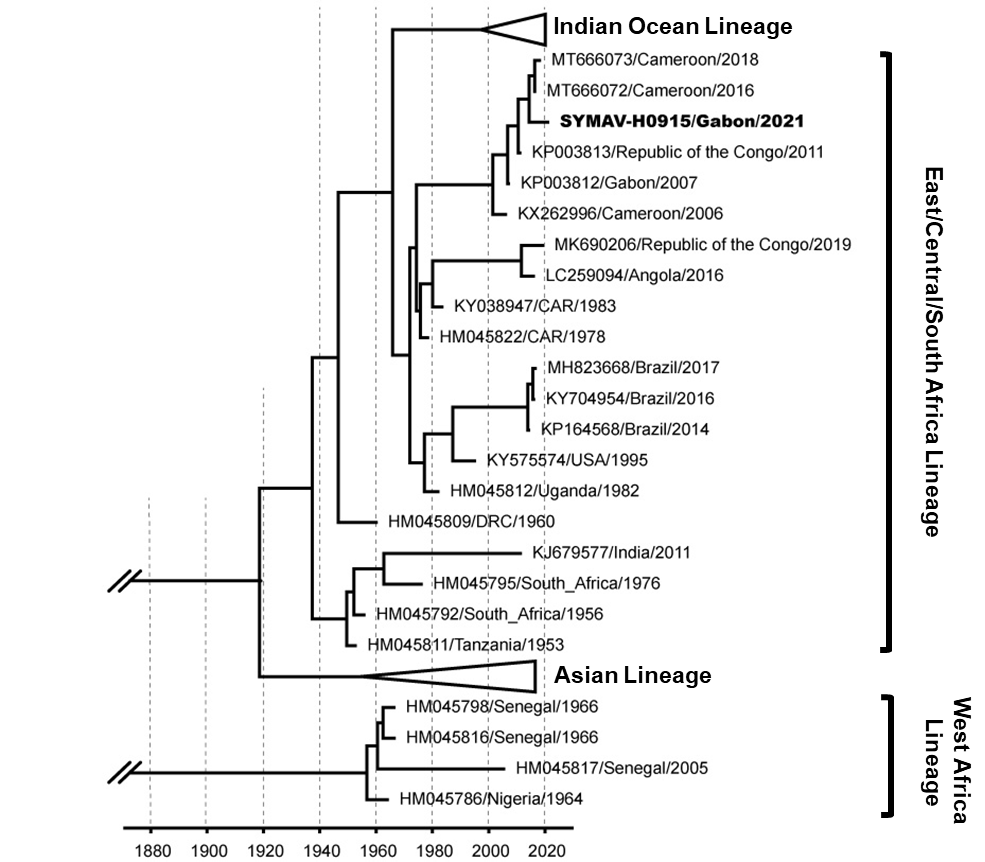


**Figure S3.** Phylogenetic analysis of the near full-length envelope 1 sequence of CHIKV. A time-scaled Bayesian maximum clade credibility tree was inferred using all African strains available. For better visualization of sequence positions, several clusters were collapsed and shown as triangles. Virus lineages are shown on the right. The Gabonese strains detected in this study are shown in bold.


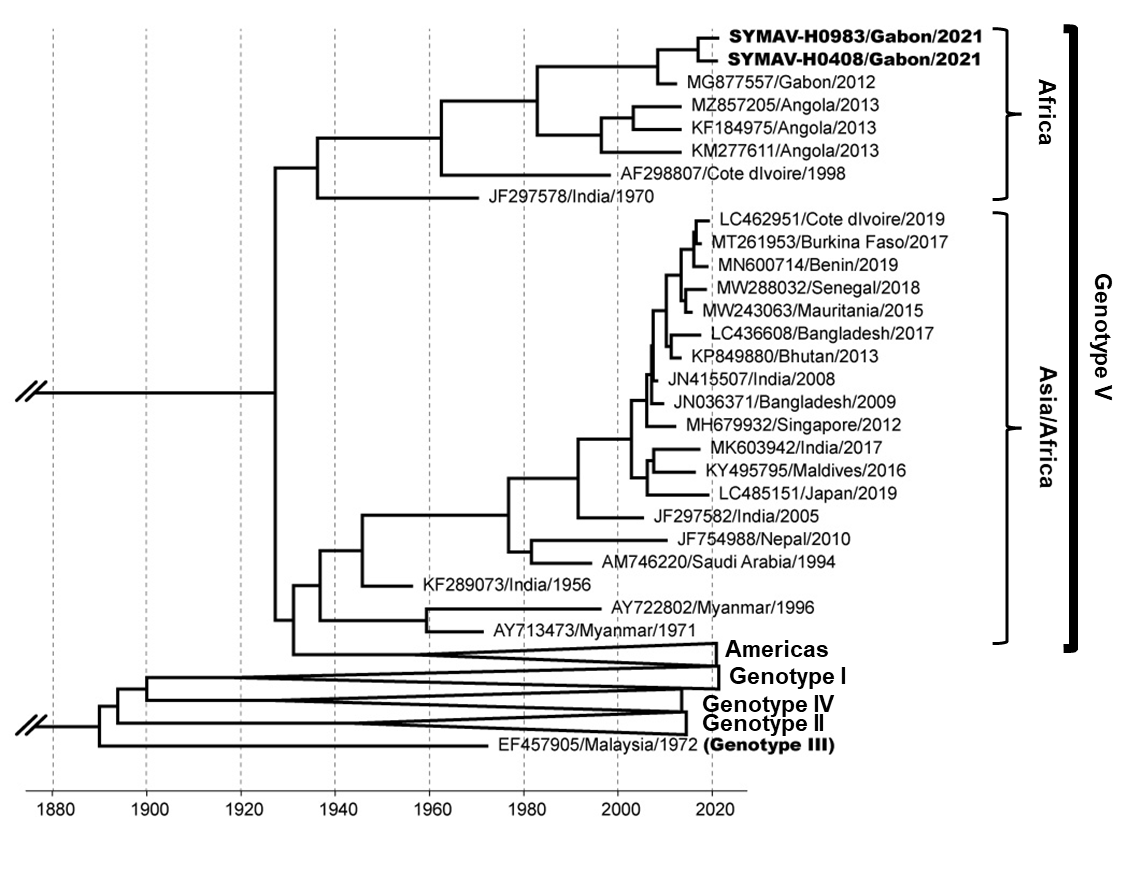


**Figure S4.** Phylogenetic analysis of the full-length envelope sequence of DENV-1. A time-scaled Bayesian maximum clade credibility tree was inferred using all African strains available. For better visualization of sequence positions, several clusters were collapsed and shown as triangles. Virus genotype are shown on the right. The Gabonese strains detected in this study are shown in bold.


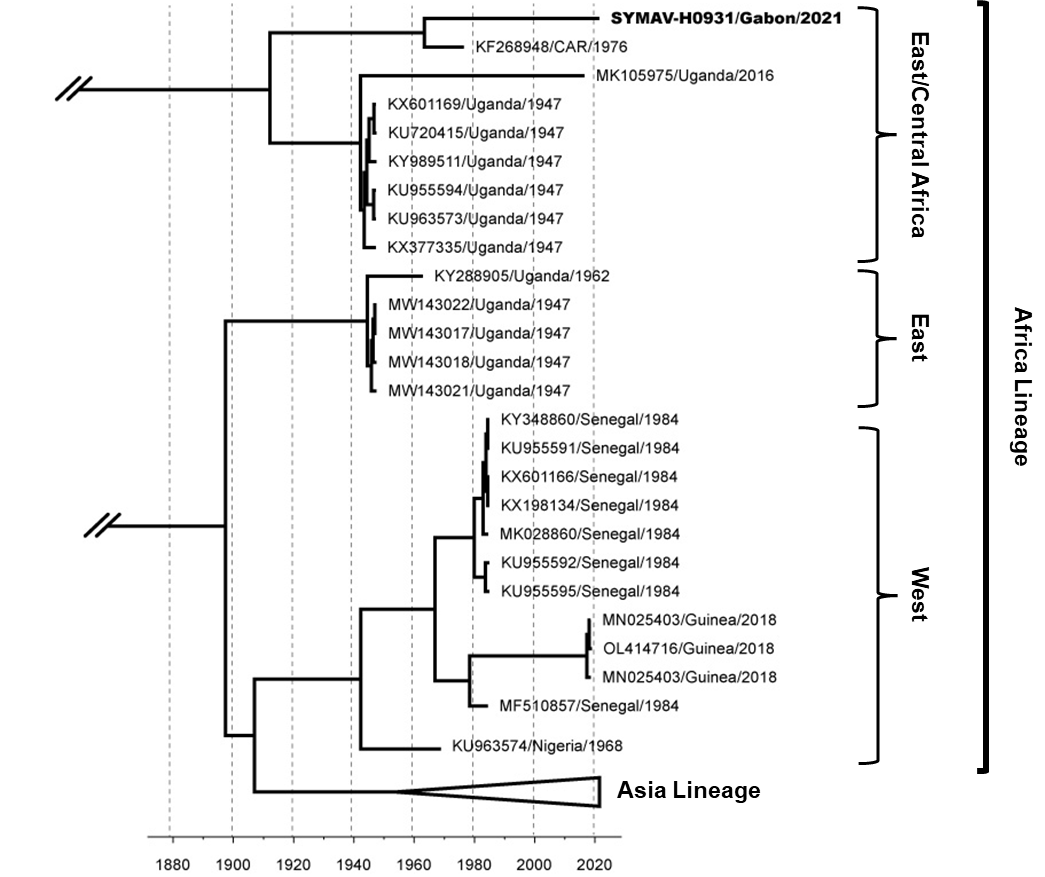


**Figure S5.** Phylogenetic analysis of the full-length non-structural protein 3 sequence of ZIKV. A time-scaled Bayesian maximum clade credibility tree was inferred using all African strains available. For better visualization of sequence positions, Asian lineages were collapsed and shown as triangles. Virus lineages are shown on the right. The Gabonese strains detected in this study are shown in bold.


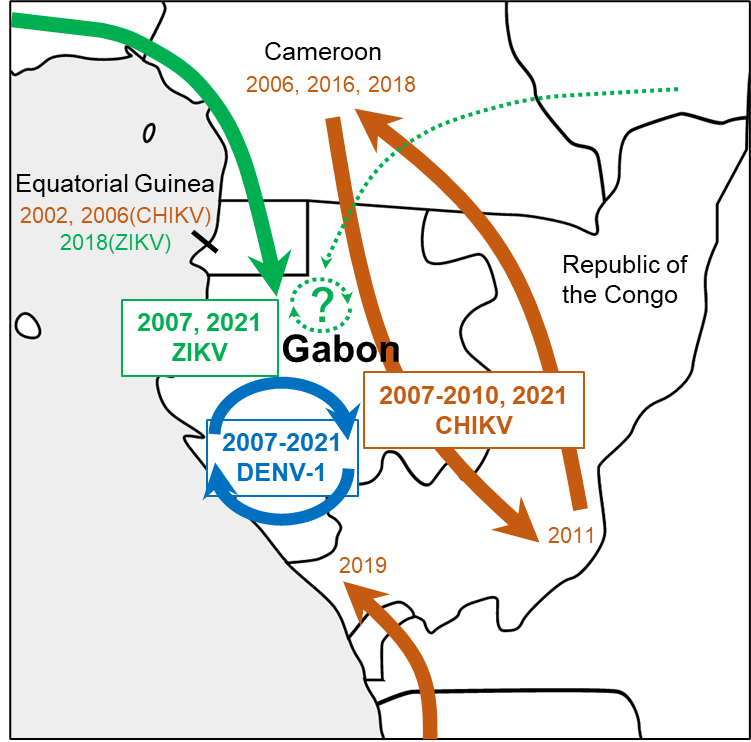


**Figure S6.** Geographical spatiotemporal dynamics of DENV-1, CHIKV and ZIKV in Central Africa. The transmission pattern is based on the phylogenetic trees shown in this study (Figure 1, 2, Supplemental Figure S1, and S3‒S5) and information on detections of the viruses (Collao et al., 2010; Fritz et al., 2019). Each colour represents DENV-1 (blue), CHIKV (brown), and ZIKV (green).

**Table S1.** List of primers and probes used in this study.

| **Name** | | **Oligonucleotide** | **Reference** |
| --- | --- | --- | --- |
| RT-qPCR | |  |  |
|  | DENG-1_CDC_8978_Fw | CAAAAGGAAGTCGYGCAATA | Vaughn et al., 2000 |
|  | DENG-1_CDC_9089_Rv | CTGAGTGAATTCTCTCTGCTRAAC | Vaughn et al., 2000 |
|  | DENV1-CDC-Probe | CATGTGGYTGGGAGCRCGC | Vaughn et al., 2000 |
|  | DENG-2_CDC_1639_Fw | CAGGCTATGGCACYGTCACGAT | Vaughn et al., 2000 |
|  | DENG-2_CDC_1716_Rv | CCATYTGCAGCARCACCATCTC | Vaughn et al., 2000 |
|  | DENV2-CDC-Probe | CTCYCCRAGAACGGGCCTCGACTTCAA | Vaughn et al., 2000 |
|  | DENG-3_CDC_742_Fw | GGACTRGACACACGCACCCA | Vaughn et al., 2000 |
|  | DENG-3_CDC_815_Rv | CATGTCTCTACCTTCTCGACTTGYCT | Vaughn et al., 2000 |
|  | DENV3-CDC-Probe | ACCTGGATGTCGGCTGAAGGAGCTTG | Vaughn et al., 2000 |
|  | DENG-4_CDC_919_Fw | TTGTCCTAATGATGCTRGTCG | Vaughn et al., 2000 |
|  | DENG-4_CDC_1007_Rv | TCCACCYGAGACTCCTTCCA | Vaughn et al., 2000 |
|  | DENV4-CDC-Probe | TYCCTACYCCTACGCATCGCATTCCG | Vaughn et al., 2000 |
|  | CHIKV_2540_Fw | CAGTGCGGCTTCTTCAATATG | This study |
|  | CHIKV_2685_CDS_Rv | CGCATTTTGCCTTCGTAATG | This study |
|  | CHIKV-2588-Probe | AACATCTGCACYCAAGTGTACCACAAAAGT | This study |
|  | ZIKA_1985_Fw | GGAACTCCACACTGGAACAACA | This study |
|  | ZIKA_2136_CDS_Rv | CCCTTTGCACCATCCATCTC | This study |
|  | ZIKA-2027-P1-Bz | AAGGACGCACATGCCAAAAGGCAA | This study |
|  | ZIKA-2027-P2-Af | AAGGAYGCCCACGCCAAGAGGCAA | This study |
| RT-PCR | |  |  |
|  | DENV1-E_820_F | AGACACCCAGGATTTACGGTGATAGCC | This study |
|  | DENV1-E_1410_R | TGGTCTCATTTCCCACCTGGTGC | This study |
|  | DENV1-E_1182_F | GTGGAAGAACAAGACGCRAACTTTG | This study |
|  | DENV1-E_2575_R | ATGCCTTCCCAATGGCTGCTG | This study |
|  | CHIKV-E1_9850_F | AAGCCCTTATTCCGCTGGCAGC | This study |
|  | CHIKV-E1_10905_R | GCCTGAGAGTATGGCACATGTACC | This study |
|  | CHIKV-E1_10882_F | GTACATGTGCCATACTCTCAGGCAC | This study |
|  | CHIKV-E1_11268_R | AGCACCACGATTAGAATCAGTGCTGC | This study |
|  | ZIKV-NS3_4470_F | TCTGGTGGAGGAAGATGGTCCACC | This study |
|  | ZIKV-NS3_5970_R | CTGAGCTCCAGGCTCTCTCTGG | This study |
|  | ZIKV-NS3_5755_F | CGGGTCATACAACTCAGCAGGAAGAC | This study |
|  | ZIKV-NS3_6630_R | GATGGTCTCTAGGGTCTCCGGCAG | This study |
|  | ZIKV-E_940_F | CTTGGTYATGATAYTGYTGATTGCCCC | This study |
|  | ZIKV-E_2341_R | TGCATGTCCACCGCCATYTG | This study |
|  | ZIKV-E-1725_F | GGMTCCCAGCACAGTGGGATGA | This study |
|  | ZIKV-E-2922_R | GRGGGGAGTCAGGATGGTACTTGTA | This study |

**Supplementary References**

Abe H, Ushijima Y, Bikangui R, Zoa-Assoumou S, Ondo GN, Manouana GP, More A, Skarwan E, Yali-Assy-Oyamli Y, Ndeboko B, Myrabelle Avome Houechenou R, Djoba Siawaya JF, Lell B, Adegnika AA, Yasuda J. Unrecognized introduction of SARS-CoV-2 variants of concern to Central Africa: Import and local transmission of B.1.1.7 in Gabon in the very early stage of the variant spread to the African continent. J Med Virol. 2021;93(10):6054-58.

Collao X, Negredo AI, Cano J, Tenorio A, Ory Fd, Benito A, Masia M, Sánchez-Seco MP.Different lineages of Chikungunya virus in Equatorial Guinea in 2002 and 2006. Am J Trop Med Hyg. 2010;82(3):505-7.

Fritz M, Taty RT, Portella C, Guimbi C, Mankou M, Leroy EM, Becquart P. Re-emergence of chikungunya in the Republic of the Congo in 2019 associated with a possible vector-host switch. Int J Infect Dis. 2019;84:99-101.

Quick J, Grubaugh ND, Pullan ST, Claro IM, Smith AD, Gangavarapu K, Oliveira G, Robles-Sikisaka R, Rogers TF, Beutler NA, Burton DR, Lewis-Ximenez LL, de Jesus JG, Giovanetti M, Hill SC, Black A, Bedford T, Carroll MW, Nunes M, Alcantara LC Jr, Sabino EC, Baylis SA, Faria NR, Loose M, Simpson JT, Pybus OG, Andersen KG, Loman NJ. Multiplex PCR Method for MinION and Illumina Sequencing of Zika and Other Virus Genomes Di-rectly from Clinical Samples. Nat. Protoc. 2017;12(6):1261-76.

Vaughn DW, Green S, Kalayanarooj S, Innis BL, Nimmannitya S, Suntayakorn S, Endy TP, Raengsakulrach B, Rothman AL, Ennis FA, Nisalak A. Dengue viremia titer, antibody response pattern, and virus serotype correlate with disease severity. J Infect Dis. 2000;181(1):29.
